# A Case Report of Bouveret Syndrome

**DOI:** 10.7759/cureus.107659

**Published:** 2026-04-24

**Authors:** Quitzia M Rentería Fonseca, Edwin Omar Fragoza Aguillares, Jenifer Alejandra Sanchez Bravo, Rodolfo Maza Gomez

**Affiliations:** 1 General Surgery, Hospital General Dr. Aquiles Calles Ramírez, Instituto de Seguridad y Servicios Sociales de los Trabajadores del Estado (ISSSTE), Tepic, MEX

**Keywords:** bouveret syndrome, enterolithotomy, gallstone ileum, intestinal pseudo-obstruction, spontaneous pneumobilia

## Abstract

The present study aimed to describe the diagnosis and surgical management of Bouveret syndrome, an uncommon cause of intestinal obstruction. We report a case of a 74-year-old woman presenting with clinical features consistent with intestinal obstruction, in whom clinical evaluation, laboratory tests, and contrast-enhanced abdominopelvic computed tomography were performed to establish the diagnosis. Surgical treatment was subsequently carried out by exploratory laparotomy and enterotomy for gallstone extraction.

Computed tomography revealed pneumobilia and the presence of an ectopic gallstone associated with intestinal obstruction, findings consistent with Rigler’s triad. Laparotomy with stone extraction and primary closure was performed without cholecystectomy. The patient had a favorable postoperative course, with restoration of bowel function and no immediate complications. Bouveret syndrome should be considered in elderly patients presenting with intestinal obstruction and a history of cholelithiasis. Computed tomography is essential for diagnosis, and surgical management represents a safe and definitive option when endoscopic treatment is not feasible or has failed.

## Introduction

Bouveret syndrome is a rare variant of gallstone ileus caused by the migration and impaction of a large gallstone through a bilioenteric fistula, most commonly a cholecystoduodenal fistula, resulting in gastric outlet obstruction [1]. Gallstone ileus accounts for 1-4% of the causes of mechanical intestinal obstruction; however, duodenal involvement is exceptional, occurring in only 1-3% of cases. This condition accounts for approximately 2-3% of all gastrointestinal obstructions due to gallstones, and only 0.3-5% of patients with cholelithiasis develop bilioenteric fistulas. A higher prevalence has been documented in elderly women, with a female-to-male ratio of 9:1 and a mean age of presentation in the eighth decade of life [2].

Reported risk factors include a history of cholelithiasis, stones larger than 2-8 cm, female sex, and age over 60 years; approximately 43-68% of patients present with a history of recurrent biliary colic, jaundice, or acute cholecystitis. Following repeated inflammatory episodes, the gallbladder adheres to the gastrointestinal tract, and mechanical pressure from the gallstone promotes ischemic necrosis of the wall, leading to fistula formation and migration of the stone into the intestinal lumen [3].

Bilioenteric fistulas are predominantly cholecystoduodenal (68%). Small stones are usually passed spontaneously; however, large stones and anatomical abnormalities predispose to obstruction. In some cases, spontaneous distal migration of the gallstone may occur, a phenomenon described as a “tumbling” or “rolling” obstruction [4].

Clinical suspicion is essential for diagnosis. Plain abdominal radiography is diagnostic in only approximately 21% of cases. Rigler’s triad (pneumobilia, intestinal obstruction, and ectopic gallstone) is highly suggestive of gallstone ileus, although it is detected in a low percentage of radiographs because many stones are radiolucent; in contrast, it can be observed in up to 80% of computed tomography scans [5].

Computed tomography is the diagnostic modality of choice, as it allows identification of the obstruction, ectopic gallstone, and sometimes the bilioenteric fistula. Upper gastrointestinal endoscopy may identify the stone in approximately 69% of cases and the fistulous opening less frequently, and may also offer a potential therapeutic approach. However, 20-40% of cases are diagnosed intraoperatively [6].

Due to advanced age and multiple comorbidities, Bouveret syndrome is associated with a morbidity of approximately 60% and a mortality ranging from 12% to 30%. Treatment options include endoscopic extraction, lithotripsy, and surgery. The following two main surgical strategies are described: isolated enterolithotomy or a one-stage procedure involving cholecystectomy and closure of the biliodigestive fistula [7]. The prognosis is favorable with timely diagnosis and appropriate treatment [1].

## Case presentation

A 74-year-old female patient presented with a history of systemic arterial hypertension, treated with losartan, hydrochlorothiazide, and acetylsalicylic acid. Her surgical history includes bilateral tubal ligation. She also has a history of chronic calculous cholecystitis and has been undergoing preoperative evaluation since 2020.

Her current illness began four days prior to admission, characterized by generalized abdominal pain, rated 8/10 on the visual analog scale (VAS), continuous, without radiation or identifiable triggering factors, and with partial relief after analgesic administration. It was associated with abdominal distension, absence of bowel movements, nausea without vomiting, and decreased intestinal transit.

On physical examination, the patient was hemodynamically stable, with a distended abdomen, high-pitched (struggling) peristalsis, and tenderness to deep palpation predominantly in the left iliac fossa, without signs of peritoneal irritation. Initial laboratory findings showed leukocytosis with neutrophilia, elevated C-reactive protein levels, and urinary findings suggestive of urinary tract infection, as summarized in Table 1.

**Table 1:** Patient laboratory findings on admission.

Parameters	Results	Normal values
Hemoglobin	9.7 g/dL	11.6-15.5 g/dL
Leukocytes (WBC)	14,000 cells/mm³	4,000-10,000 cells/mm³
Neutrophils	89%	40-70%
Platelets	242,000 cells/mm³	150,000-400,000 cells/mm³
C-reactive protein (CRP)	211 mg/L	<5 mg/L
Glucose	137 mg/dL	70-100 mg/dL
Total cholesterol	90 mg/dL	<200 mg/dL
Albumin	4.3 g/dL	3.5-5.0 g/dL
Urinalysis		
Leukocytes	30 per high-power field (HPF)	0-5 per high-power field (HPF)
Leukocyte esterase	Positive	Negative
Nitrites	Positive	Negative
Bacteria	Numerous	None
Erythrocytes	0-2 per HPF	0-2 per HPF

**Figure 1:**
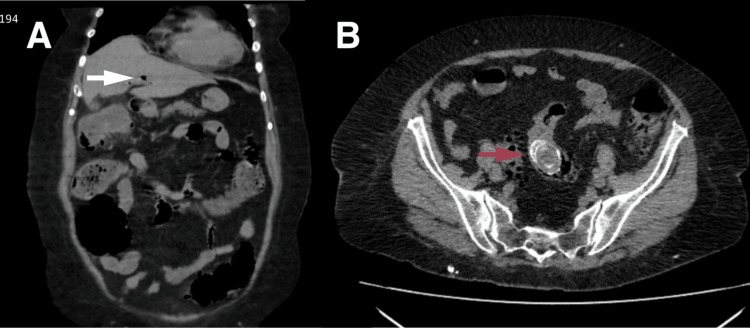
Abdominopelvic computed tomography. (A) Axial section showing gas within the intrahepatic biliary tree (pneumobilia) (white arrow). (B) Axial section identifying a hyperdense intraluminal image corresponding to an ectopic gallstone impacted in the sigmoid colon (red arrow). The findings are consistent with Rigler’s triad and are suggestive of gallstone ileus (Bouveret syndrome).

**Figure 2:**
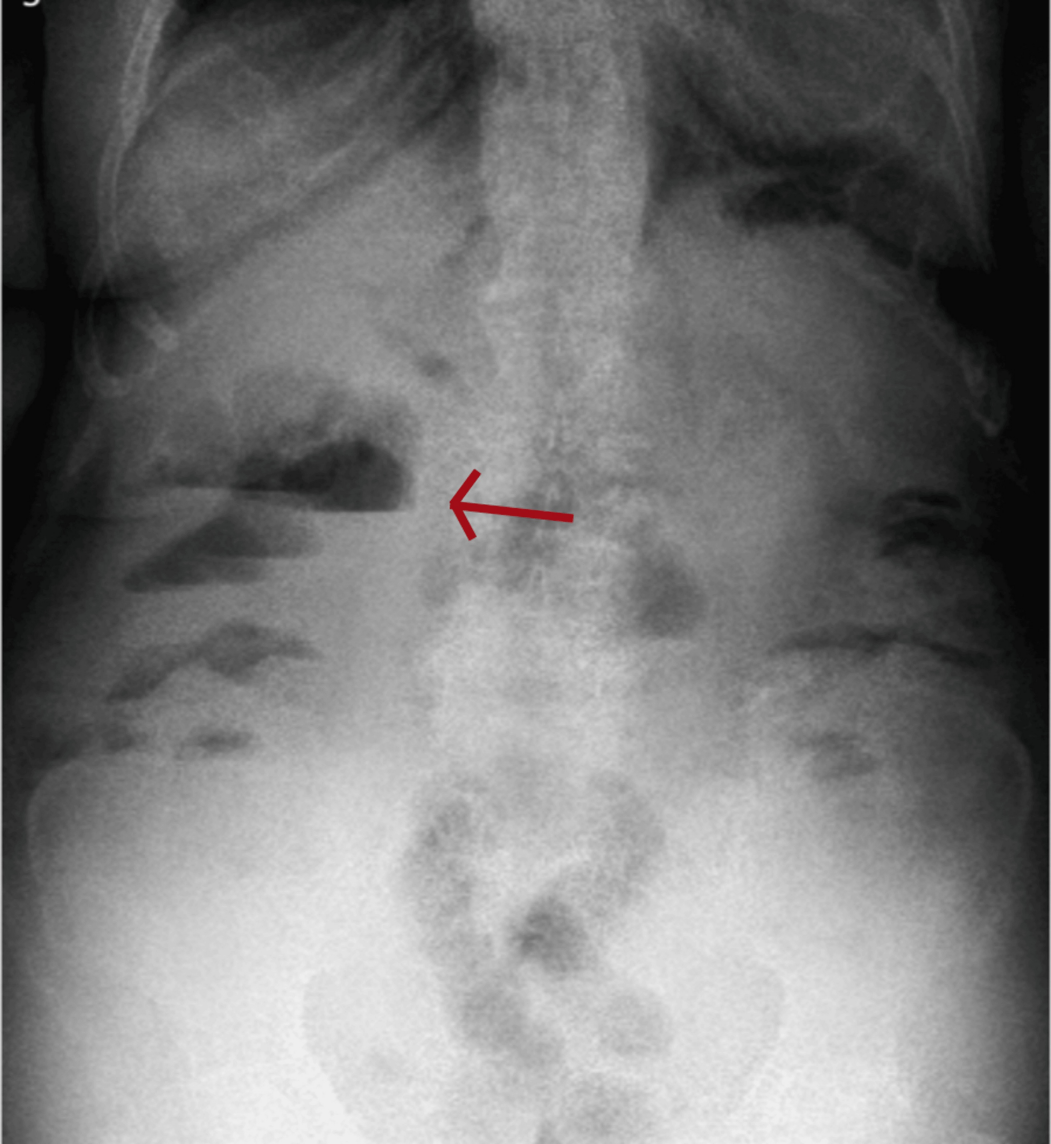
Plain abdominal radiograph in the upright position. Dilatation of small bowel loops with multiple air-fluid levels (red arrow).

A contrast-enhanced abdominopelvic computed tomography scan was performed, revealing hepatomegaly, the presence of gas in the biliary tract (pneumobilia), and mild dilation of the biliary tree. The gallbladder appeared reduced in size. Additionally, a colonic inflammatory process with probable obstruction was identified, associated with a calcified image measuring approximately 6×2 cm located within the sigmoid colon (Figures 1A, 1B). Plain abdominal radiography showed distension of small bowel loops with multiple air-fluid levels (Figure 2). Imaging findings were suggestive of Rigler’s triad (intestinal obstruction, pneumobilia, and ectopic gallstone), consistent with gallstone ileus and Bouveret syndrome.

Results

The patient underwent an exploratory laparotomy. During the procedure, an indurated, mobile intraluminal mass measuring approximately 6×2 cm was identified in the sigmoid colon, without evidence of adhesions to adjacent structures. Due to its mobility, the stone was manually advanced distally toward the descending colon as an intentional intraoperative maneuver to facilitate a safer surgical approach.

**Figure 3:**
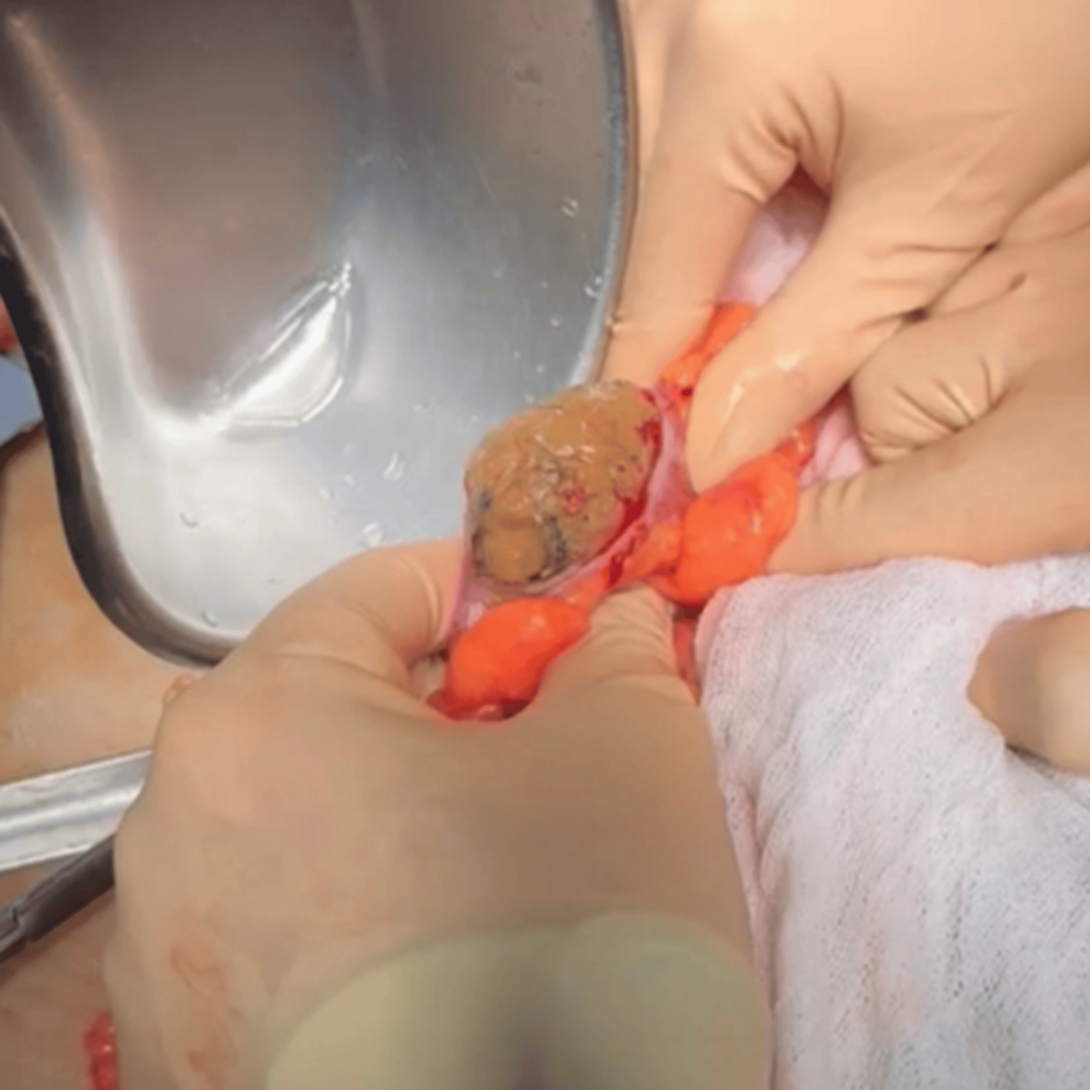
Intraoperative colotomy showing an intraluminal gallstone. A longitudinal colotomy in the descending colon is observed, through which an intraluminal gallstone is exteriorized, responsible for the mechanical intestinal obstruction.

**Figure 4:**
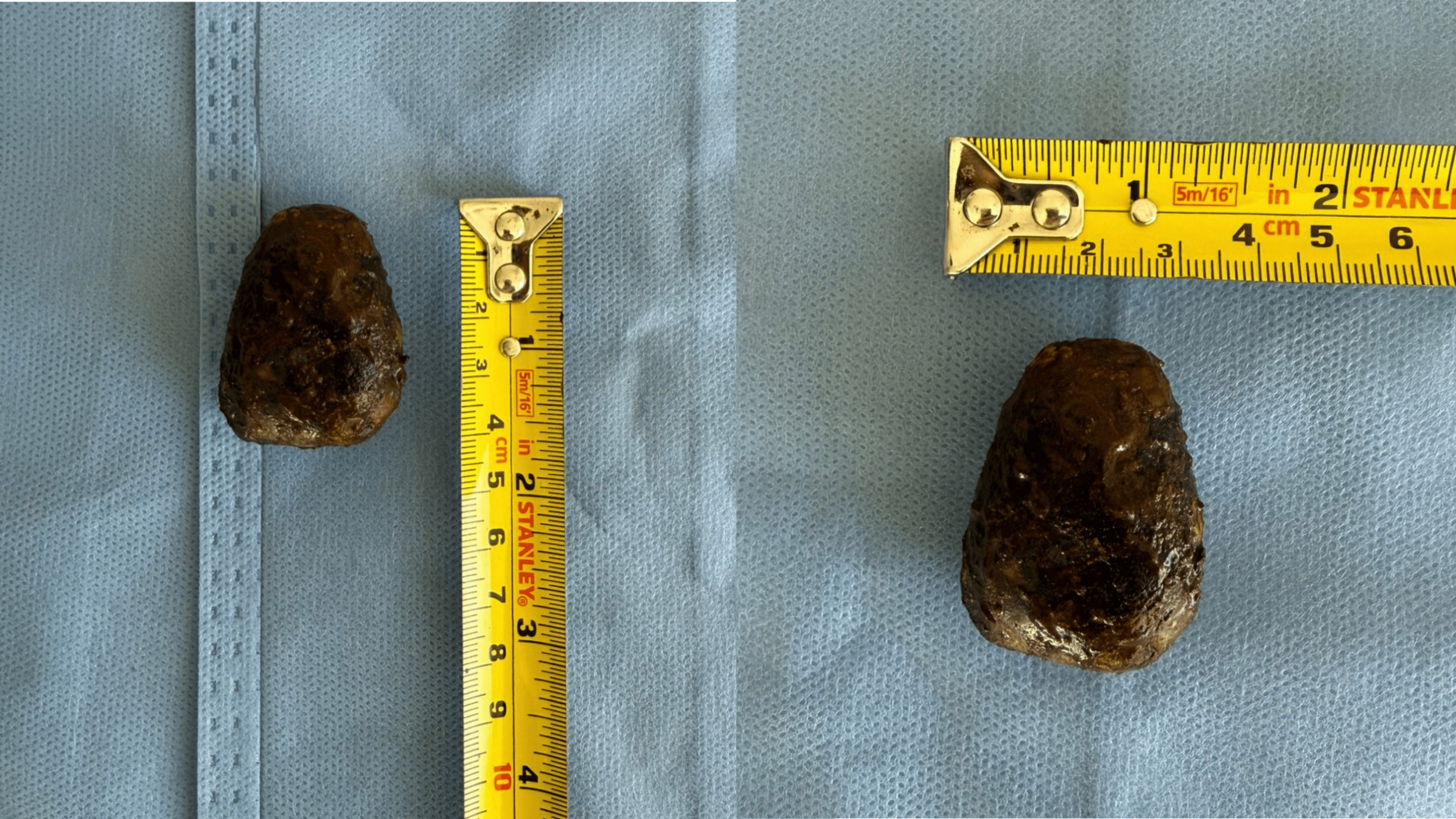
Extracted gallstone causing intestinal obstruction. Large gallstone following extraction, placed on the surgical field with a reference scale, measuring approximately 6×2.5 cm.

Subsequently, a longitudinal colotomy of approximately 4 cm was performed at the level of the descending colon through which a gallstone was extracted (Figure 3). After extraction, the colotomy was closed transversely using the Heineke-Mikulicz technique in two layers, with no evidence of luminal stenosis or vascular compromise, and without intraoperative complications. In Figure 4, the surgical specimen is shown, corresponding to a gallstone measuring 6×2.5 cm.

Cholecystectomy and closure of the cholecystoenteric fistula were not performed due to the presence of a scleroatrophic gallbladder and the absence of signs of active inflammation or associated complications; therefore, a one-stage approach was not indicated.

The patient had an uneventful postoperative course and was discharged on postoperative day three once the risk of immediate complications had subsided. At the six-month follow-up, the patient remained asymptomatic with no evidence of complications or recurrence.

## Discussion

Bouveret syndrome is defined as an uncommon clinical variant of gallstone ileus, characterized by obstruction of the gastric outlet or duodenum caused by the migration of a gallstone through a bilioenteric fistula [1,2]. Although gallstone ileus accounts for 1-4% of the causes of mechanical intestinal obstruction, the specific duodenal localization (Bouveret) is exceptional, occurring in only 1-3% of these cases [2,3]. It accounts for approximately 2-3% of gastrointestinal obstructions due to gallstones, and only 0.3-5% of patients with cholelithiasis develop bilioenteric fistulas [2]. In the clinical case analyzed, the patient presented with an obstruction in the sigmoid colon, which, although classified in the text within the spectrum of Bouveret syndrome, technically represents a form of distally located gallstone ileus [5,6].

The patient, a 74-year-old woman with a history of chronic cholelithiasis, closely matches the demographic profile described in the literature. A marked female predominance has been documented, with a 9:1 ratio relative to men and a mean age at presentation in the eighth decade of life [3]. Key risk factors present in this case include age over 60 years and a history of recurrent gallstone disease, which predispose to adhesion formation and ischemic necrosis of the gallbladder wall, facilitating fistula formation [1]. Approximately 43-68% of patients present with a history of recurrent biliary colic, jaundice, or acute cholecystitis [3].

Clinical suspicion is challenging due to the nonspecific nature of symptoms. In this report, abdominopelvic computed tomography was the definitive diagnostic tool, identifying Rigler’s triad as follows: pneumobilia, intestinal obstruction, and ectopic gallstone. This literature emphasizes that while plain radiography detects this triad in only 21% of cases, computed tomography (CT) increases diagnostic sensitivity to up to 80%, establishing it as the gold standard. Ultrasound may help exclude concomitant biliary pathology and evaluate the gallbladder or fistula [5]. It is noteworthy that although imaging showed a calcified lesion measuring 4×4 cm, the extracted stone measured 6×2.5 cm, highlighting the importance of considering the actual size of the stone for surgical planning.

Regarding the therapeutic strategy, the choice lies between isolated enterolithotomy and one-stage surgery. The management of Bouveret syndrome and gallstone ileus may be either endoscopic or surgical; although endoscopic treatment is considered the first-line approach, it frequently fails due to the large size of the stones, as likely occurred with the 6 cm calculus in the present case [4,5]. Concerning the surgical approach, the following two main strategies are described in the literature: isolated enterolithotomy, which involves resolution of the obstruction alone, and one-stage surgery, which combines enterolithotomy with cholecystectomy and fistula closure.

In this case, the surgical team opted for isolated enterolithotomy without cholecystectomy. This decision is consistent with literature recommendations for elderly patients or those with comorbidities, as combined surgery is associated with higher postoperative mortality. Given that the gallbladder was scleroatrophic and showed no signs of acute inflammation, the conservative approach allowed favorable recovery and restoration of intestinal transit without the complications associated with complex biliary surgery in a fragile patient.

The prognosis for these patients is favorable with early diagnosis. However, the literature reports mortality rates ranging from 12% to 30%, largely due to the advanced age of affected individuals [1]. The decision not to close the cholecystoenteric fistula in this patient carries a theoretical risk of recurrence or biliary sepsis, but in the context of a scleroatrophic gallbladder, the benefits of reducing operative time generally outweigh these risks.

## Conclusions

Bouveret syndrome should be considered an important differential diagnosis in elderly patients presenting with intestinal obstruction, especially if they have a known history of cholelithiasis. To confirm this condition, computed tomography is established as the diagnostic tool of choice, given its high sensitivity for identifying Rigler’s triad, which consists of pneumobilia, intestinal obstruction, and the presence of an ectopic gallstone. In the present case, computed tomography demonstrated findings consistent with intestinal obstruction and an ectopic gallstone, supporting the diagnosis.

Although initial management may be attempted endoscopically, surgical intervention represents the safest and most definitive option when this approach is not feasible or proves insufficient due to the size of the stone. Specifically, in patients with multiple comorbidities or advanced age, isolated enterolithotomy without simultaneous cholecystectomy or fistula closure is recommended, as this strategy reduces postoperative mortality and promotes a favorable clinical course with restoration of intestinal transit. In the present case, enterolithotomy without cholecystectomy or fistula repair was performed due to intraoperative findings and the patient’s clinical condition, resulting in an uneventful recovery. Finally, the prognosis of this condition is favorable as long as timely diagnosis and appropriate surgical treatment are ensured, preventing critical complications such as severe dehydration or intestinal wall perforation. In this case, the patient remained asymptomatic at six-month follow-up with no evidence of complications or recurrence.
